# From Routine to Crisis: The Impact of COVID‐19 Pandemic on Antibiotic Consumption in Iran

**DOI:** 10.1002/hsr2.70161

**Published:** 2024-10-29

**Authors:** Satar Rezaei, Mohammad Bazyar, Sina Ahmadi, Abdolvahed Khodamoradi

**Affiliations:** ^1^ Research Center for Environmental Determinants of Health, Health Institute Kermanshah University of Medical Sciences Kermanshah Iran; ^2^ Health Management and Economics Department, Faculty of Health Ilam University of Medical Sciences Ilam Iran; ^3^ Social Development & Health Promotion Research Center, Health Institute Kermanshah University of Medical Sciences Kermanshah Iran; ^4^ Department of Health Planning Petroleum Industry Health Organization Tehran Iran

**Keywords:** antibiotics, COVID‐19, Iran

## Abstract

**Background and Aims:**

The COVID‐19 pandemic has significantly impacted the healthcare sector, influencing patients, providers, and the overall system. This study evaluates how the pandemic affected antibiotic prescriptions among 44 million Iranians insured by the Social Security Organization (SSO).

**Methods:**

In this quasi‐experimental study, we utilized monthly aggregated data on antibiotic prescriptions per 1000 individuals insured by the SSO. We employed a single‐group interrupted time series analysis (ITSA) over a period of 72 months, from March 20, 2016 to February 19, 2020 for the pre‐pandemic phase, and from February 20, 2020 to March 20, 2022, for the during‐pandemic period. Additionally, we conducted a multiple‐group ITSA to assess the differential impact of the pandemic on antibiotic consumption between the direct (SSO‐owned medical centers) and indirect (other private or public centers contracted by the SSO) sectors.

**Results:**

The study revealed a 30% reduction in the monthly average antibiotic consumption rate when comparing the during‐pandemic period to pre‐pandemic usage across the total sector. The results from the single‐group ITSA indicated that the mean antibiotic consumption per 1000 individuals at baseline was 1664. Following the onset of the pandemic, there was a significant reduction in antibiotic prescriptions, dropping to 484 per 1000 individuals (*p* ≤ 0.001) in the first month. However, during the pandemic period, antibiotic prescriptions exhibited insignificant monthly increases, averaging 10.7 per 1000 insured individuals. The multiple‐group ITSA revealed that both sectors experienced a decline in antibiotic prescriptions after the outbreak of COVID‐19. Notably, the indirect sector demonstrated a greater reduction, with a decrease of 187 prescriptions per 1000 insured individuals in the first month following the pandemic's onset.

**Conclusions:**

Our study found a significant reduction in antibiotic consumption. Further research is needed to compare antibiotic use between hospitals and outpatient centers, as well as among COVID‐19 and non‐COVID‐19 patients.

## 1 Introduction

On March 11, 2020, the World Health Organization (WHO) officially declared the COVID‐19 outbreak a pandemic [1, 2, 3]. This outbreak not only disrupted the global economy, leading to the closure of numerous businesses, but it also had a profound impact on healthcare systems worldwide. It significantly affected healthcare utilization, access to services, and medical operations across the globe [4]. As a result of the COVID‐19 pandemic, there was a sharp decline in in‐person healthcare services, such as outpatient visits, while the use of telehealth services saw a significant rise [5, 6, 7]. This shift was primarily driven by patients' concerns about potential exposure to COVID‐19 in healthcare settings and the cancellation of elective procedures, including surgeries [7, 8]. These measures also led to a decline in the occurrence of various other infectious diseases, not just COVID‐19, in both inpatient and outpatient settings [9, 10, 11]. For example, in areas such as Europe and the United States, where COVID‐19 infection rates were elevated, there was a notable and abrupt decrease in antibiotic prescriptions within the community following the introduction of restrictions aimed at controlling the pandemic [12, 13, 14].

Interpreting the underlying data presents challenges, as it encompasses a range of factors and varies in terms of infrastructure and the effectiveness of preventive measures, including public health constraints, healthcare access, social policies, rates of COVID‐19 infections, the availability of telehealth services and even environmental factors [15] such as the quality of weather, air pollution and humidity, and temperature of the region [16, 17]. While some studies indicated a decline in community antibiotic prescriptions following the implementation of COVID‐19 restrictions in early 2020, others noted a rapid increase in antibiotic usage during the initial wave of the pandemic [18, 19]. This rise can be partly attributed to the presence of other bacterial infections in COVID‐19 patients, necessitating antibiotic treatment, as well as disruptions to antibiotic stewardship programs [18, 20, 21, 22]. Additional factors contributing to the increased antibiotic consumption during the pandemic included the absence of effective treatment strategies, overlapping symptoms with pneumonia, and concerns about the risk of hospital‐acquired infections [23, 24]. For example, a systematic review analyzing 130 studies found that approximately 78% of COVID‐19 patients were prescribed antibiotics [25].

Other studies have indicated varying trends based on healthcare settings. For example, Andrews et al. noted that in England, antibiotic prescriptions decreased in the community during the pandemic in 2020, while there was a rise in antibiotic use among hospital admissions [18]. These findings also underscored the necessity for more data on antibiotic consumption in low‐income countries [26]. Consequently, additional research is essential to achieve a thorough understanding of the factors influencing antibiotic usage during the pandemic across different contexts [18]. Having reliable, precise and in time information and analysis about the abrupt rise or reduction in antibiotics consumption due to pandemic, such as COVID‐19 and reasons behind it are so important in terms of rational antibiotic consumption and lessons which can have for controlling antibiotic resistance and effects of self‐medications [27, 28]. Iran presents a unique case for examining the impact of significant events like the COVID‐19 pandemic on antibiotic consumption, as it was one of the first countries to experience a high number of new COVID‐19 cases and faced substantial mortality rates in the Middle East for an extended period, although these figures may be underestimated [29, 30, 31]. It is also worth to mention that according to previous studies, antibiotics consumption in Iran is three times more than OECD countries [32]. This makes investigating the impact of COVID‐19 pandemic on antibiotic consumption more important.

Similar to many other countries, Iran implemented common preventive measures to curb transmission rates, including the closure of public spaces such as universities, schools, and places of worship, as well as promoting handwashing, social distancing, wearing masks, and using hand sanitizer immediately following the onset of the pandemic [33, 34]. The absence of telemedicine services in Iran likely disrupted regular access to healthcare and affected antibiotic consumption. Conversely, the surge in new COVID‐19 cases and high mortality rates among infected patients, combined with the fear surrounding an unfamiliar disease and the lack of effective treatments or vaccines, likely contributed to an increased use of antibiotics as an additional remedy.

While the impact of COVID‐19 on antibiotic consumption has been extensively studied in high‐income countries [35, 36, 37], there is a significant lack of data from developing countries, particularly Iran. This gap is critical, as healthcare systems in developing nations face unique challenges that may influence antibiotic prescribing practices. In this study, we evaluated antibiotic consumption among over 50% of Iran's population covered by the Social Security Organization (SSO) over a 6‐year period, including 4 years before the pandemic and 2 years during the pandemic. As there would be always new pandemics in future, although it is not clear when, being prepared for potential future pandemics with similar impacts on society is necessary [38]. So comparing and combining of various findings from wide range of contexts can help policymakers to make reliable decisions based on scientific information and devise and adopt evidence‐based policies at least to manage the consumption of antibiotics.

## 2 Methods

This quasi‐experimental study utilized antibiotic prescription data from the SSO at the national level in Iran. The SSO is one of the three basic social health insurance organizations that provide coverage for both formal and informal private sector workers. As a nongovernmental entity, the SSO operates 78 hospitals, 312 general and specialized clinics, and 5 outpatient surgery centers, making it a key provider of healthcare services alongside public and private sectors. It offers free healthcare services directly to its beneficiaries through these facilities (referred to as the direct sector) and also by contracting the public and private hospitals and health centers (known as the indirect sector) [39, 40]. As of 2021, approximately 44 million individuals, representing over half of Iran's total population, were covered by the SSO [41].

The data was obtained from the SSO's insurance information system, covering the period from March 2016 to 2022 (47 months before the pandemic and 25 months following its onset in Iran). To examine changes in antibiotic consumption in detail, the data was analyzed separately for the direct, indirect, and total sectors. The first confirmed case of COVID‐19 in Iran occurred on February 19, 2020, marking the beginning of the outbreak in the country. Consequently, the periods from March 20, 2016 to February 19, 2020 and from February 20, 2020 to March 20, 2022, were designated as the pre‐pandemic and during‐pandemic periods, respectively.

We examined how the COVID‐19 pandemic affected the number of antibiotics prescribed per 1000 insured individuals by the SSO. Therefore, the main outcome variable was the number of antibiotics prescribed per 1000 insured individuals. The independent variable was time, represented by aggregated monthly data on antibiotics prescribed to insured individuals through the SSO. Our study included three time‐based independent variables in single group Interrupted Time Series Analysis (ITSA) (see Equation 1) and seven time‐based independent variables in multiple group ITSA (see Equation 2).

To examine changes in antibiotic consumption before and during the COVID‐19 pandemic, we applied ITSA, which is particularly useful for evaluating interventions that occur at specific points in time and affect entire populations; it has been employed to assess various public health interventions and events, including health programs and sector reforms [42, 43] as well as unplanned events like the COVID‐19 pandemic on population health trends [44, 45, 46]. The flexibility of ITSA makes it a valuable quasi‐experimental methodology for determining the influence of both planned interventions and unforeseen events when randomized controlled trials are not feasible. In our study, we utilized both single and multiple group ITSA to evaluate the impact of the COVID‐19 pandemic on the rate of antibiotics consumed per 1000 insured individuals in the SSO in Iran, with single group ITSA assessing the pandemic's effects on outcome variables in both the direct and indirect sectors as well as the total population, while multiple group ITSA was used to compare the impacts between the direct and indirect sectors [47, 48]. We employed the following model for the single group ITSA [49]:

(1) $$Y_{t}=\beta_{0}+\beta_{1}\times time_{t}+\beta_{2}\times COVID19_{t}+\beta_{3}\times time\;\text{during}\;COVID19_{t}+\varepsilon_{t}$$

Where *Y*_*t*_ is the outcome variable of antibiotic consumption in month t. *time*_*t*_ is a continuous time trend variable coded from 1 to the total number of months. *COVID*19_*t*_ is a binary indicator variable capturing the pre‐pandemic period (coded 0) and pandemic period (coded 1). *time* during *COVID*19_*t*_ is a continuous variable indicating the number of months after the start of the pandemic, coded from 1 to the number of pandemic months. *β*_0_ estimates the baseline level of the outcome at time zero, *β*_1_ estimates the pre‐pandemic trend, *β*_2_ estimates the level change during the pandemic, and *β*_3_ estimates the change in trend after COVID‐19 compared to the pre‐pandemic trend.

The multiple groups ITSA was used to investigate the effects of the COVID‐19 pandemic on antibiotic consumption in the direct sector versus the indirect sector. The following segmented regression model was used for the analysis [48]:

(2) $$Y_{t}=\beta_{0}+\beta_{1}T_{t}+\beta_{2}X_{t}+\beta_{3}T_{t}X_{t}+\beta_{4}Z+\beta_{5}{\text{ZT}}_{t}+\beta_{6}{\text{ZX}}_{t}+\beta_{7}{\text{ZT}}_{t}X_{t}+e_{t}$$

Y_*t*_ is the outcome variable (e.g., antibiotic consumption); *T*_*t*_ is the time since start of study (continuous); *X*_*t*_ is the binary variable representing pre/during pandemic periods (0 = pre, 1 = during); *Z* is the binary variable representing direct versus indirect sectors (0 = direct, 1 = indirect); *T*_*t*_X_*t*_, Z*T*_*t*_, ZX_*t*_ Z*T*_*t*_X_*t*_ are interaction terms; *β*_0_ is the intercept (starting level of Y in direct sector); *β*_1_ is the pre‐pandemic slope (trend in Y over time in direct sector); *β*_2_ is the level change during pandemic (change in Y in direct sector during pandemic); *β*_3_ is the difference between pre/during pandemic slopes (change in trend in Y during pandemic in direct sector); *β*_4_ is the difference in baseline Y between direct and indirect sectors; *β*_5_ is the difference in pre‐pandemic trends between direct and indirect sectors; *β*_6_ is the difference in level change during pandemic between direct and indirect sectors; *β*_7_ is the difference in pre/during pandemic slope change between direct and indirect sectors and *e*_*t*_ is the error term.

Newey West standard errors were used in the regression models to correct for autocorrelation and heteroscedasticity. The actest command was used to identify the optimal lag length to control for autocorrelation in each outcome variable. To examine stationary status of the outcome variables, the augmented Dickey−Fuller test was used. Stationary status was confirmed based on *p*‐values less than 0.05 for each outcome variable [48]. All data analyses were performed using Stata version 17. A *p*‐value less than 0.05 was considered statistically significant.

## 3 Results

Table 1 presents information about the amount of changes in antibiotics consumption before and during outbreak of COVID‐19 pandemic. It shows that on average there was 30% reduction in consumption of antibiotics during the pandemic compared to before that in total sector.

**Table 1:** Descriptive statistics of antibiotic consumption per 1000 insured by the Social Security Organization (SSO) in Iran: Pre‐pandemic (March 2016 to January 2020) and during the pandemic (February 2020 to March 2022).

Outcome variables		Before the pandemic	During the pandemic	Change %	*Z*	*p* value
		Mean (standard deviation)	Mean (standard deviation)			
Antibiotic consumption per 1000 insured	Direct sector[Link], ^a^	515.5 (145.3)*	383.6 (86.7)	−25.6	4.3	< 0.001
	Indirect sector*	1116.9 (174.6)	755.5 (141.5)	−32.4	6.2	< 0.001
	Total*	1632.4 (275.5)	1139.1 (214.5)	−30.2	5.8	< 0.001

*Note:* *0.001.

^a^ The SSO provides health services to insured patients in two main ways: direct and indirect sectors. In the direct sector, healthcare facilities owned by SSO, including hospitals, clinics, pharmacies, rehabilitation centers, and so forth provide free treatment and medicines to patients. In the indirect sector, the SSO has contracts with hospitals/medical centers affiliated with medical universities, private hospitals, labs, physicians' offices, and so forth to provide care to insured patients. Patients often prefer the direct sector because it is free. But this causes long wait times. Patients who can afford it may go to the indirect sector for quicker access.

**Figure 1:**
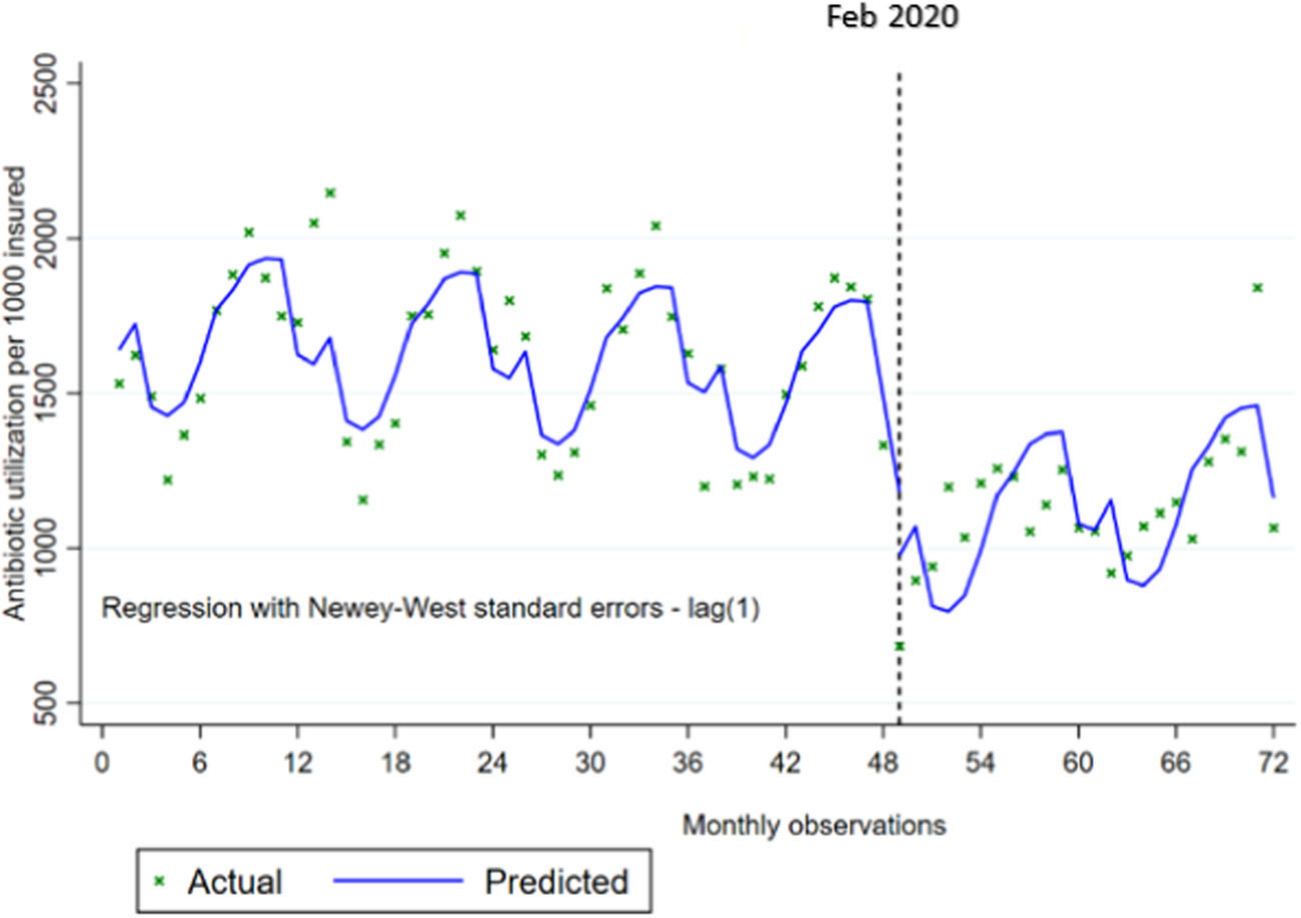
Single group Interrupted Time Series Analysis (ITSA) on the effect of the COVID‐19 pandemic on antibiotic consumption per 1000 insured individuals by the Social Security Organization (SSO) in Iran: pre‐pandemic (March 2016 to January 2020) and during the pandemic (February 2020 to March 2022). Note: The pandemic outbreak was in February 2020 in Iran.

Table 2 shows the results of the single group analysis for the changes in antibiotic consumption during COVID‐19 pandemic in SSO as a whole (combining direct and indirect sectors). At the starting level, the number of antibiotics consumed prescribed was 1664 per 1000 insured in the total sector. There was a significant declining trend before pandemic and number of antibiotics reduced by 3.7 per 1000 insured monthly. In the first month after the pandemic onset, a substantial reduction occurred in the antibiotic prescription which was 484 per 1000 individuals. The slope of longer changes during the pandemic shows that the number of antibiotics followed an ascending trend, increasing 10.7 per 1000 insured each month, although it was not significant. Figure 1 visually illustrates the changes in antibiotic consumption before and during the pandemic for total sector.

**Table 2:** Results of single group Interrupted Time Series Analysis (ITSA) on the effect of the COVID‐19 pandemic on antibiotic consumption per 1000 insured individuals by the Social Security Organization (SSO) in Iran: pre‐pandemic (March 2016 to January 2020) and during pandemic (February 2020 to March 2022).

	Coefficient	Newey‐West standard error	95% confidence interval
Mean value at the baseline (*β*_0_)	1664.8*	24.11	1526.58 to 1803.09
Pre‐trend (*β*_1_)	−3.7**	−2.26	−7.06 to −0.43
During‐level change (*β*_2_)	−483.9*	−3.71	−745.31 to −222.61
During‐trend change (*β*_3_)	10.7***	1.28	−6.01 to 27.51
Model significance: *F*, (*p* value)	19 (< 0.001)		

*0.001, **0.05 and ***not significant.

The results of the multiple groups of ITSA have been presented in Table 3, comparing the changes in antibiotic consumption in direct sector relative to indirect sector of SSO. As indicated in the table, the average number of antibiotic prescriptions in hospitals and health facilities affiliated with SSO (direct sector) was 532 per 1000 insured. The slope of changes was descending but negligible, less than 1 antibiotic per 1000 individuals over time prior the pandemic. In the first month of the pandemic, there was a major reduction in number of antibiotics in the direct section which was 213 per 1000 insured but it started to rise 9.0 antibiotics per 1000 persons monthly.

**Table 3:** Results of multiple groups Interrupted Time Series Analysis (ITSA) on the effect of the Covid‐19 pandemic on antibiotic consumption per 1000 insured individuals by the Social Security Organization (SSO) between the direct and indirect sectors in Iran: Pre‐pandemic (March 2016 to January 2020) and during pandemic (February 2020 to March 2022).

			Coefficient	Newey‐West standard error	95% confidence interval
Direct sector	Before the pandemic	Mean value at the baseline (*β*_0_)	532.83*	57.41	419 to 646
		Pre‐trend (*β*_1_)	−0.75***	1.80	−4.32 to 2.81
	During the pandemic	During‐level change (*β*_2_)	−213.46*	46.35	−305 to −121
		During‐trend change (*β*_3_)	9.01*	2.68	3.7 to 14.3
Indirect sector relative to direct sector^a^	Before the pandemic	Pre‐level difference (*β*_4_)	587.60*	88.73	412 to 763
		Pre‐trend difference (*β*_5_)	0.60***	3.21	−5.7 to 6.9
	During the pandemic	During‐level difference (*β*_6_)	−187.15**	101.51	−387.9 to 13.6
		Change in slope difference pre to during (*β*_7_)	−5.34***	5.88	−16.9 to 6.3
Model significance: *F*, (*p* value)			67.04 ( < 0.001)*		

*0.001,**0.1 and ***not significant.

a The SSO provides health services to insured patients in two main ways: direct and indirect sectors. In the direct sector, healthcare facilities owned by SSO, including hospitals, clinics, pharmacies, rehabilitation centers, and so forth provide free treatment and medicines to patients. In the indirect sector, the SSO has contracts with hospitals/medical centers affiliated with medical universities, private hospitals, labs, physicians' offices, so forth to provide care to insured patients. Patients often prefer the direct sector because it is free. But this causes long wait times. Patients who can afford it may go to the indirect sector for quicker access.

Before the pandemic, there was a significant difference between the direct and indirect sectors in terms of antibiotic consumption. The indirect sector had a baseline level of antibiotic use that was 587.6 per 1000 insured people higher than that of the direct sector. However, the slope difference between the two sectors was not significant before the pandemic.

**Figure 2:**
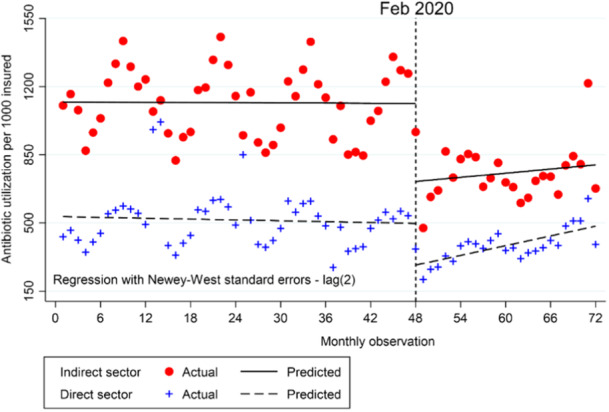
Multiple group Interrupted Time Series Analysis (ITSA) on the effect of the COVID‐19 pandemic on antibiotic consumption per 1000 insured individuals by the Social Security Organization (SSO) between the direct and indirect sectors in Iran: pre‐pandemic (March 2016 to January 2020) and during pandemic (February 2020 to March 2022). Note: The pandemic outbreak was in February 2020 in Iran. The SSO provides health services to insured patients in two main ways: direct and indirect sectors. In the direct sector, healthcare facilities owned by SSO, including hospitals, clinics, pharmacies, rehabilitation centers, and so forth provide free treatment and medicines to patients. In the indirect sector, the SSO has contracts with hospitals/medical centers affiliated with medical universities, private hospitals, labs, physicians' offices, and so forth to provide care to insured patients. Patients often prefer the direct sector because it is free. But this causes long wait times. Patients who can afford it may go to the indirect sector for quicker access.

Right after the outbreak of COVID‐19, according to the $\beta_{6}$, the immediate level change during the pandemic between direct and indirect sectors was −187 per 1000 insured. It means that the reduction in antibiotic consumption in the indirect sector was 187 per 1000 more than what happened in the direct section, which was significant at the level of 10%. The difference in pre/during pandemic slope change between direct and indirect sectors was also negligible and not significant. Figure 2 visually compares the changes in antibiotic consumption before and during the pandemic in both direct and indirect sections simultaneously.

## 4 Discussion

In our study, we found that the outbreak of the COVID‐19 pandemic in Iran led to a significant decrease in overall antibiotic consumption among a large population covered by the SSO. Specifically, we observed a 30% reduction in total antibiotic dispensing in the months following the pandemic. This trend was also observed in other countries, although at different levels. The degree of decrease in antibiotic utilization rates during the COVID‐19 pandemic was as follows: China (2.8%), Canada (26.5%), Scotland (34%), Jordan (5.5%), and British Columbia (12.79%) [50].

The results of the single group analysis showed a substantial reduction in the antibiotic prescription which was 484 per 1000 individuals. The slope of longer changes during the pandemic shows that the number of antibiotics followed an ascending trend, increasing 10.7 each month, although it was not significant. A part of this reduction might be due to a decline in healthcare services utilization, which was also observed in other studies. Another study in Iran showed that on average the number of emergency department and outpatients visits per 10,000 population in the west of Iran reduced by 191.65 and 168.57, respectively [51]. So the reduction of visits directly reduced the number of antibiotics prescribed by the physicians. Likewise, other studies also found that right after the COVID‐19 pandemic, the number of outpatient visits at public clinics almost reduced by 40% [52, 53]. The lack of telemedicine services in Iran may have further contributed to this trend. Potential factors contributing to this reduction may include shifts in patient behavior and disruptions in healthcare services, both of which could have influenced antibiotic consumption patterns. On the contrary, a multi‐center retrospective study in Iran examined the use of 16 broad‐spectrum antibiotics among 43,791 hospitalized COVID‐19 patients during the first 6 months of the pandemic. The study revealed significant antibiotic use even in the absence of confirmed bacterial co‐infections among COVID‐19 patients [54]. These contradictory findings necessitate investigating antibiotic consumption separately in outpatient and inpatient settings.

To prevent the spread of COVID‐19, Iran, like other countries, implemented strict policies such as staying at home, mask‐wearing, physical distancing, closure of kindergartens, schools, universities, jobs, religious places and ceremonies, washing hands, and travel limitations [55, 56]. These measures not only reduced COVID‐19 transmission but also decreased the transmission of other endemic respiratory infections. A systematic review by Dadras et al. showed that adherence to health protocols to prevent COVID‐19 can reduce the incidence of infectious diseases such as influenza, pneumonia, and tuberculosis [57]. Similar findings were reported from other regions. In Australia, restrictions in mobility within the country resulted in reduced person‐to‐person transmission of respiratory and gastrointestinal pathogens. Indeed, following the implementation of restrictions, dramatic falls were reported in the incidence of virologically confirmed and clinical episodes of influenza, respiratory syncytial virus (RSV), and infectious gastroenteritis [9]. Antibiotic prescriptions for upper respiratory tract infections decreased by 71% during the early part of the pandemic, while those for urinary tract infections only decreased by 3% [58]. Another study showed that antibiotics recommended for respiratory tract infections showed large reductions (range 51%−69%), whereas those for non‐respiratory infections remained unchanged [9].

The study conducted a multiple group ITSA to compare antibiotic consumption between the direct and indirect sections of SSO. The findings revealed that during the first month of the study, healthcare providers in health centers other than SSO facilities prescribed an average of 587 more antibiotics per 1000 insured, which was statistically significant. However, there was a negligible difference in trends between the two sections before the pandemic. Following the outbreak of COVID‐19, both sections experienced a reduction in antibiotic prescriptions, but the indirect sector showed a greater reduction of 187 per 1000 insured. Various factors may have contributed to the difference before the pandemic, such as the higher number of visits and health service utilization per 1000 insured in the indirect section and the greater availability of specialists who can prescribe more sophisticated antibiotics. Additionally, SSO has a limited pharmacopeia and does not cover branded medicines, which may influence healthcare providers' choices. Finally, while healthcare services are free for beneficiaries in the direct section, SSO has implemented initiatives to manage and control unnecessary patient demands, including restricting protocols for medication and antibiotic prescriptions by physicians.

It is worth noting that non‐SSO facilities experienced a greater reduction in antibiotic consumption during the COVID‐19 outbreak. This may be attributed to the fact that the Ministry of Health and Medical Education (MoHME) in Iran primarily utilized public hospitals and government resources to treat COVID‐19 patients, resulting in a higher reduction in health service utilization in these centers. People may have been hesitant to visit these hospitals for non‐COVID‐19‐related illnesses. On the other hand, SSO beneficiaries may have found their facilities safer to use and did not reduce their healthcare utilization in SSO facilities as much as they did in other sections.

## 5 Strengths and Limitations of the Study

The study's major strength lay in its utilization of extensive recorded data for over 42 million SSO beneficiaries. We employed robust interrupted time series methods, including single and multiple models, to estimate the population‐level changes in antibiotic usage. The ITSA design, based on a segmented regression approach, is a powerful quasi‐experimental design that enables the evaluation of the overall impact of the COVID‐19 pandemic on antibiotic prescribing trends. This study is the first of its kind to investigate changes in antibiotic consumption at a national level in Iran, a developing country, using big data. It can provide valuable insights into the effects of the pandemic on antibiotic prescription in low‐middle‐income countries, as emphasized in systematic reviews. A key strength of this study is the use of extensive time series data to analyze changes in antibiotic consumption before and after the pandemic. We examined 47 months of pre‐pandemic data and 25 months of post‐pandemic data. This allows us to paint a more complete picture compared to other studies that utilized fewer time points, a noted limitation. However, the study had certain limitations. The first significant limitation of this study is the reliance on aggregated monthly data for antibiotic consumption. This aggregation may obscure variations in prescribing patterns that occur within individual months, potentially masking short‐term trends or fluctuations in antibiotic use. Additionally, aggregated data do not allow for a detailed analysis of differences between inpatient and outpatient antibiotic consumption, which may have varied significantly during the pandemic. These factors could limit the generalizability of our findings and warrant caution in interpreting the results. This limitation arises from our reliance on aggregated data for antibiotic consumption, which hindered a separate analysis of changes in the inpatient and outpatient sectors. Such an analysis could have enhanced our understanding of treatment‐seeking behaviors in both hospitals and outpatient facilities, thereby informing the development of targeted policies. Additionally, we analyzed changes per 1000 insured instead of per 1000 visits, which would have been more appropriate, especially in multiple models. However, this was not possible as some beneficiaries use non‐contracted physicians and health providers for visits, and their visit data is not delivered to SSO (but antibiotics that they prescribe would be finally recorded), which makes the data unclear and complicated for analysis. Another limitation is that government policies during the pandemic, such as quarantine and physical distancing, could have influenced healthcare consumption due to changes in patient and provider behaviors. However, since these policies typically lasted less than 1 month and our data were aggregated monthly, it was not feasible to analyze this aspect due to the lack of specific data. Finally, it is important to acknowledge potential limitations related to the accuracy and completeness of the data obtained from the SSO's records. Variations in data quality may impact the reliability of our findings and should be considered when interpreting the results.

## 6 Policy Recommendations

We observed a significant decrease in antibiotic consumption during the COVID‐19 pandemic. It is unclear whether this reduction has led to an overall improvement or deterioration in public health. However, it does warrant examination in terms of rational medication use and antibiotic resistance. We need to be prepared for future epidemics and understand their impact on the increased or decreased use of antibiotics, as well as the lessons that can be drawn regarding antibiotic resistance. If this reduction in antibiotic consumption has not harmed health in general and is found that previous consumption had been due to self‐medication or over‐prescription by physicians, then fundamental actions must be taken regarding the rational use of antibiotics in the post‐COVID era [59]. It is recommended that insurance organizations, which routinely collect and record vast amounts of data on the consumption of various healthcare services including antibiotics, establish mechanisms now to monitor antibiotic use accurately, distinguishing between inpatient and outpatient settings, respiratory and non‐respiratory cases, as well as different diseases. This will enable more precise analyses in the future, which will be highly effective and vital from both policy‐making and evidence‐based medical perspectives. Moreover, it is suggested that, in line with findings from other studies, the government focus on strengthening the healthcare system—including medical equipment and human resources, vast vaccination campaigns, as well as public health measures to better prepare for similar pandemics [60, 61, 62]. This is crucial because the benefits of using antibiotics for treating airborne viral infections have not been proven [63].

## 7 Conclusion

Our study revealed that a significant reduction in antibiotic consumption occurred in Iran by the COVID‐19 pandemic and the way it changed the general healthcare services utilization in health provision system and all restrictions that people faced in their routine lives. However, we could not differentiate the potential differences that might happen between hospitals due to dealing with a high number of COVID‐19 patients and outpatient centers. We also could not provide precise analysis to compare changes in antibiotic use between COVID‐19 and non‐COVID‐19 patients, between respiratory and non‐respiratory infectious diseases, or at least separately for antibiotics with the highest consumption. Further studies are essential to analyze the situation and develop context‐based policies for managing antibiotic consumption in future similar scenarios. Specifically, future investigations could focus on longitudinal tracking of antibiotic consumption in the post‐pandemic period to evaluate long‐term impacts on prescribing patterns and resistance trends. Additionally, conducting more granular analyses that differentiate between various types of antibiotics and their usage in different settings—such as respiratory versus non‐respiratory infections—would yield valuable insights into treatment practices and help inform targeted interventions.

## Author Contributions

**Satar Rezaei, Mohammad Bazyar, Sina Ahmadi, and Abdolvahed Khodamoradi:** conceptualization. **Satar Rezaei, Mohammad Bazyar, Sina Ahmadi, **and **Abdolvahed Khodamoradi:** methodology. **Satar Rezaei and Mohammad Bazyar:** formal analysis. **Satar Rezaei:** writing–original draft preparation. **Mohammad Bazyar:** writing–review and editing. All authors have read and agreed to the published version of the manuscript.

## Ethics Statement

The research protocol was reviewed and approved by the Research Deputy of Kermanshah University of Medical Sciences, with the approval number IR.KUMS.REC.1402.533. The study protocol was also approved by the Social Security Research Institute, which granted permission to access and utilize the data for this analysis.

## Conflicts of Interest

The authors declare no conflicts of interest.

## Data Availability

The data sets generated and analyzed during the current study are available from the corresponding author upon reasonable request.
